# Aplication of Soil Productivity Index after Eight Years of Soil Reclamation with Sewage Sludge Amendments

**DOI:** 10.1007/s00267-020-01422-1

**Published:** 2021-01-18

**Authors:** Wiktor Halecki, Sławomir Klatka

**Affiliations:** Department of Land Reclamation and Environmental Development, Faculty of Environmental Engineering and Land Surveying, University of Agriculture in Krakow, Al. Mickiewicza 24-28, 30-059 Kraków, Poland

**Keywords:** Fuzzy logic, Phytoremediation, Sewage sludge amendments, Soil productivity index, Soil restoration, Technosol

## Abstract

Remediation methods are gaining acceptance as effective and inexpensive techniques used in the reclamation of degraded areas. The reclamation of post-mining sites has become important for the conservation of soil and vegetation. An assessment of potential productivity of plants based on the depth of their root zone is crucial for the validation of properties of post-mining soils. Our aim was to present soil productivity parameters that would facilitate assessment of various post-mining objects. Soil productivity index (SPI) was calculated to assess soil quality, mainly in areas degraded by hard coal mining. It is based on an equation determining the relationship between the productivity index and the physical, chemical, and hydrological properties of soil. Our study demonstrated the positive effects of enriched sewage sludge with amendments on newly formed soil and plants. The soil productivity index was 0.81, demonstrating the suitable condition of the initial soil resulting from reclamation. This parameter might be important for post-industrial reclamation, such as wasteland intended to be transformed into woodland. Considering the composition of sewage sludge amendments, it can be successfully used as an effective method of restoring and improving both the physical and chemical properties of soils, thus effectively replacing mineral fertilisers. The use of sewage sludge in soil reclamation will be an important method of managing this waste material in post-mining areas.

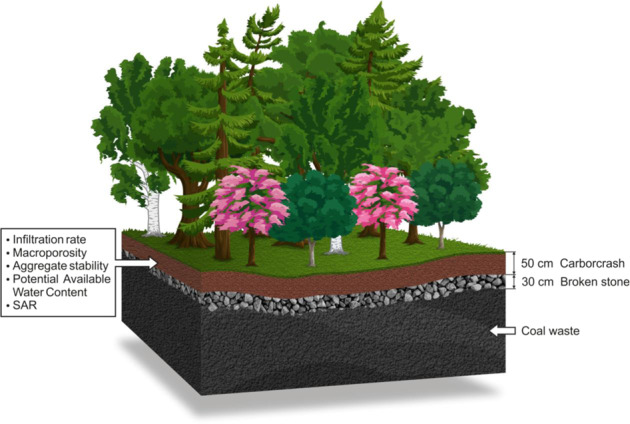

## Introduction

Modern bioremediation offers a number of solutions for effective soil reclamation (Wadgaonkar et al. 2018). Further, a land restoration strategy is a crucial aspect of managing degraded areas (Chang et al. 2011; Toktar et al. 2016). Healthy soil should be present in reclaimed areas, as this is important for maintaining the management of the degraded areas (Acton et al. 2011; Gomes 2012; Avera et al. 2015; Thijs et al. 2017). This is particularly vital for vascular plants covering those areas (Skubała 2011). Plant growth is one of the primary aims of biological reclamation (Zipper et al. 2011; Yang et al. 2012; Fields-Johnson et al. 2014; Halecki and Klatka 2017). Selecting plant species and their cultivation to ensure protection against surface erosion is a very important phase of the reclamation process (Zhao et al. 2014). Therefore, an evaluation of soil conditions of post-mining areas is essential (Bes and Mench 2008; Lors et al. 2011; Lal 2015). Proper soil structure is also vital if it is necessary to replace plant species through revegetation (replanting) (Zhang et al. 2015) and to avoid the current migration of heavy metals to soil (Pellegrini et al. 2016). The assessment of soil organic carbon (SOC) and development of grasses (Wu et al. 2010) in soil productivity studies (Klatka et al. 2014), as well as filtration coefficient and bulk density (Bi et al. 2014), are indispensable parameters for the evaluation of reclamation activities (Liu et al. 2012).

It was found that 6 years after a reclamation treatment can already be observed soil aggregates sizes appropriate for the retention of soil organic matter (Yin et al. 2016), which is considered an indicator of soil quality in reclaimed post-mining areas (Bodlák et al. 2012). Hitherto, little is known of the possible negative consequence of using sewage sludges in soils, and of their long-term behaviour. Therefore, supplementation with organic composite materials that stabilises trace elements in the soil is required (Kim et al. 2016). Further, additional treatments and various substrates and auxiliary preparations that enhance bioremediation are necessary (Singh et al. 2009). Moreover, the durability of sewage sludge, and the toxicological effects of using it for reclamation of post-mining areas, can only be assessed in long-term studies (Halecki and Klatka 2018).

In ecological engineering projects, the scale of research and performance indices should depend on uniform indicators of soil quality (Constantini et al. 2016). Evaluation of soil productivity may be performed for area reclamation purposes (Kiniry et al. 1983), or to determine the degree of soil erosion and degradation for agricultural purposes (Gantzer and McCarty 1987). The aim of this paper was to demonstrate changes in physical and chemical properties of post-mining soil after an application of Carbocrash substrate (Halecki and Klatka 2018). Post-mining areas present many challenges, mainly due to unfavourable physical and chemical properties. Considering these, our study was focused primarily on: (i) assessment of the suitability of a new mixture (composition) of sewage sludge (Carbocrash) as a substrate for reclamation of degraded soils; (ii) determination of the usefulness of soil productivity index in an evaluation of potential degrees of degradation caused by enriched sewage sludge, (iii) comparison of physical and chemical properties of initial soil at two depths where trees and shrubs were planted; (iv) establishing the main factors affecting the soil restoration process using multivariate analysis. Our research attempted to verify the following research hypothesis: Enriching post-mining area with Carbocrash substrate will improve initial soil quality.

## Material and Methods

### Study Area

**Fig. 1:**
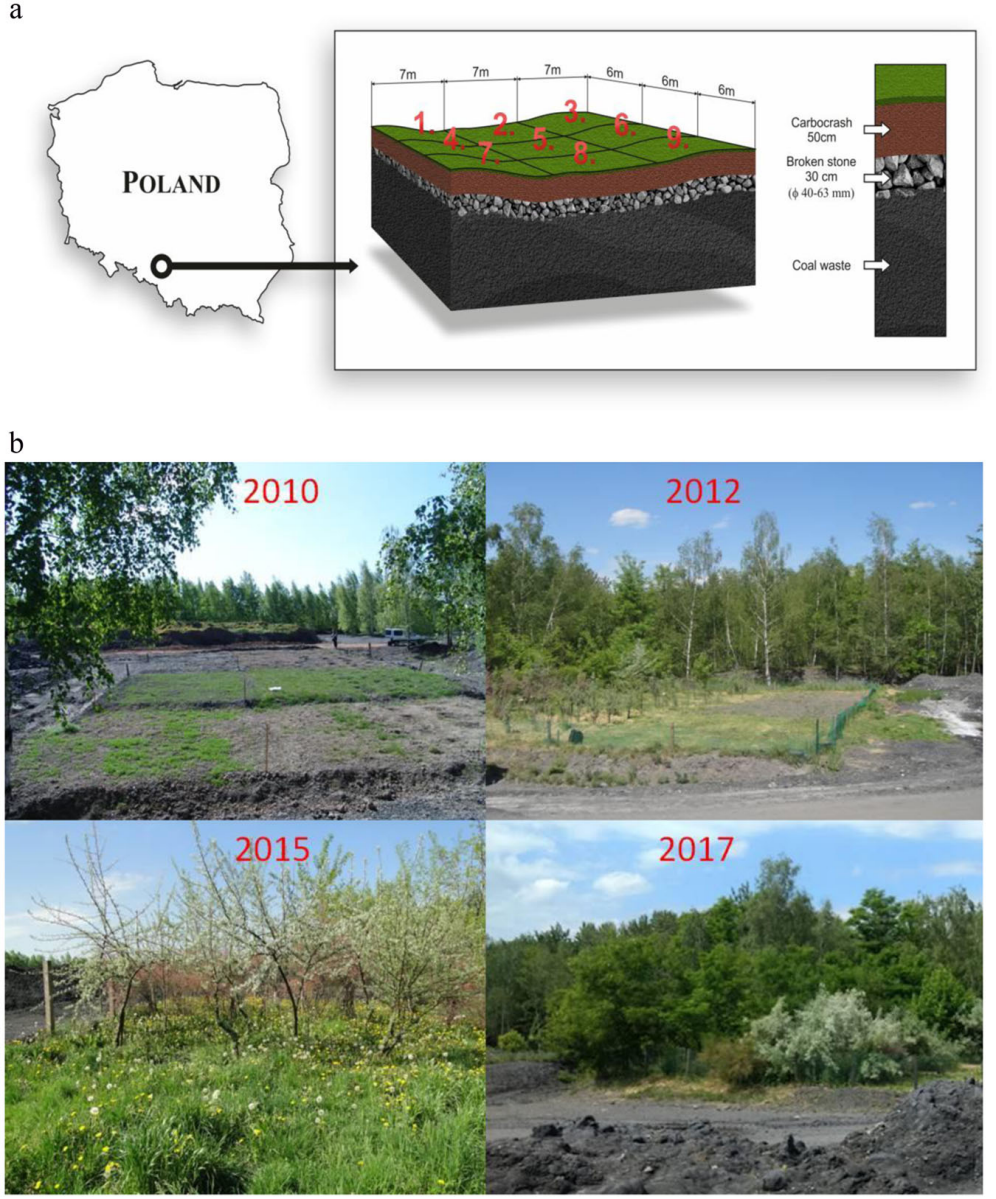
**a** Location and experimental design in research area. In order to produce a complete stand trees and shrubs were planted from plots 1 to 6. Plots 7, 8 and 9 was dedicated for native mixture of grass and herbaceous perennial plants (not described in this paper). **b** Vegetation growing on a weathered post-mine soil (Silesia Province, Poland) in consecutive years

During an 8-year field experiment conducted on a slag-heap land from post-mining activities located in the Silesia, a polluted site in Poland, the influence of the application of Carbocrash substrate (industrial waste) was investigated. The study was conducted on a mine created among slag heaps where rock raw materials (primarily coal) were stored (Fig. 1). The site was chosen because there were technical shortcomings in the relief formation by overburdening the excavated rocks, and the area lacked drainage. Prior to biological reclamation, the area was generally neglected in terms of the dumping ground management. We provided a sewage sludge-amended biosolids substrate, which we called ‘Carbocrash’ (35% by weight municipal organic sewage sludge, 30% post-flotation waste (from a coal mining plant), 20% crushed stone (angular sandstone), and 15% fly ash). The research involved experimental plots established on a drainage layer made of waste rock. This was valueless rock excavated as a gangue ore, covered with a layer of Carbocrash substrate of mean thickness 50 cm. The factor limiting proper plant development in dumping grounds is a layer of soil that is too thin, and often with insufficient nutrient content. This is why the Carbocrash layer was 50 cm thick. Total dimensions of the plots were 21 × 18 m, and they incorporated nine 7 ×6 m sectors (research areas).

### Trees and Shrubs Planted in the Post-mining Area

From 2010 to 2017 the experimental plots harboured the following species of trees and shrubs: oleaster (*Elaeagnus angustifolia*), smallflower tamarisk (*Tamarix parviflora*), and common sea-buckthorn (*Hippophae rhamnoides*) have been located in study plots 1–3, while black locust (*Robinia pseudoacacia*), silver birch (*Betula verrucosa*), maple ash (*Acer negundo*) and Douglas fir (*Pseudotsuga menziesii*) have been planted in study plots 4–6. These are the species often used in biological reclamation of post-industrial areas, owing to their high resistance to difficult growing conditions. These plant species can be considered as the most suitable for biological reclamation of brownfields with Carbocrash substrate. Crop species have been cultivated within 7–9 study plots.

When planting trees and shrubs, it is necessary to make a hole deep enough for the plant roots to develop. The holes for the trees were 40 × 40 × 50 cm, whereas for the shrub seedlings they were 30 × 30 × 40 cm. The spacing between seedlings was 1 m. Following planting, weeding was performed on the entire reclamation area for 2 consecutive years. Seedling rooting rate and viability were observed during the vegetative period in both spring and autumn, and dead seedlings were replaced in the spring. The seedlings that were partly dead (dead crowns but alive lateral shoots from the trunk or root collar) were left in place and carefully examined in the autumn. Autumn was also the season for plant treatments such as pruning the branches.

The assessment of the health of trees and shrubs on the experimental plots was conducted at the end of the growing season each year. Further, schemes of distribution of individual species are included in the research reports from previous years. The measurements were made taking into account two important parameters—height and diameter at breast height (DBH). To determine the height of the trees under investigation, a Sunnto altimeter type PM-5/1520 was used, and the DBH was measured using a Sandvik 32 cm diaphragm. Further, the thickness classes were specified to better illustrate the stand, and the distances between trees and bushes were measured. Only plants with good health were considered for inclusion in the measurements, therefore withering or broken were not considered. An inventory of trees and shrubs, and their distribution on experimental plots, were also included in previous studies (Halecki and Klatka 2018).

### Laboratory Techniques Used to Calculate the Soil Productivity Index

**Fig. 2:**
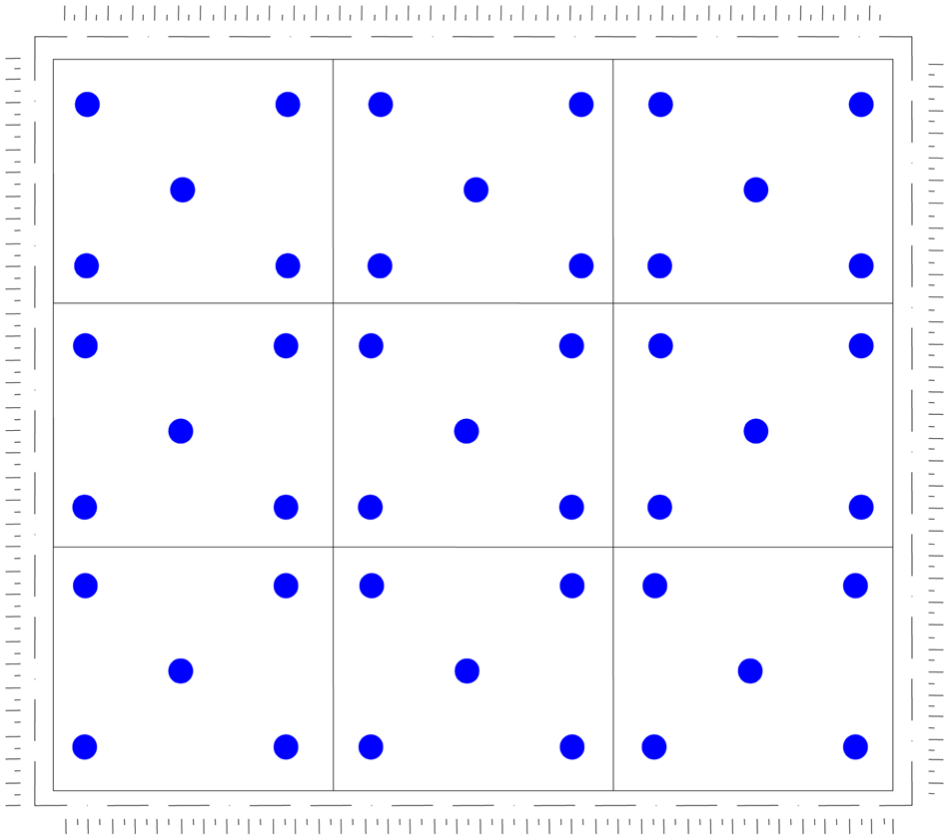
Sampling scheme in study area

Samples for laboratory tests were collected at five locations from each plot during the growing season (April–November). Forty-five samples were collected in each season. Consequently, 360 sample data were employed as the final basis for the research. The study area was divided into nine plots to facilitate easier comparison of Carbocrash substrate in each site. The sampling collection sites and the plot plan are presented in Fig. 2. Properties of the investigated substrate were determined in a series of laboratory tests. Soil texture (granulometric composition) was measured by the sieve and aerometric Bouyoucos method, as modified by Casagrande and Prószyński (Ryżak et al. 2009). Bulk density and porosity were measured by the Kopecky method (Dedousis and Bartzanas 2010). Compactness was measured with a cone penetrometer. Organic matter was determined by a modification of the Tiurin method (Ostrowska et al. 1991). Infiltration was determined by a field method of double rings (Doorenbos 1977). Filtration coefficients in the Darcy’s law-based apparatus (Baver et al. 1972) were measured with adjustable water pressure and electronic water volume readings. The potentiometry technique was applied for pH in H_2_O measurements. Specific electrical conductivity was obtained by a Slandi CM 204 conductometer in aqueous solution with a water-to-soil ratio of 5:1. The specific electrical conductivity was then converted into the content of easily soluble salts, based on a standard curve for potassium chloride. Aggregate stability was measured with a Zwick/Roell testing machine, as described by Dexter and Kroesbergen (1985). This test involved air-dry aggregates of 10–15 cm in diameter. Finally, the potential available water content was determined based on water characteristic curves prepared for pressure chambers with porous ceramic plate and parametrised van Genuchten’s equations (Wösten and van Genuchten 1988) as follows:

$$\theta = \theta _r + \frac{{\theta _S - \theta _r}}{{\left( {1 + \left| {\alpha \cdot h} \right|^n} \right)^m}}$$

where: θ—current volumetric water content in the soil (cm^3^·cm^−3^), θ_r_—air dry volumetric water content in the soil (cm^3^·cm^−3^), θ_s_—volumetric water content in the soil at full saturation (cm^3^·cm^−3^), h—suction pressure (cm), α, n, m—equation parameters determined by statistical methods.

The organic carbon content was determined by multiplying with 0.58, as soil organic matter is assumed to contain 58% of organic carbon (Pribyl 2010). Total nitrogen was determined by the modified Kjeldahl method (ISO 1995) in solutions after soil mineralisation with H_2_SO_4_ and H_2_O_2_. ICP-AES JY-238 Ultrace emission spectrometer in a solution after soil mineralisation with aqua regia was used to calculate the content of phosphorus (P), potassium (K), sodium (Na), calcium (Ca), magnesium (Mg), chromium (Cr), zinc (Zn), cadmium (Cd), copper (Cu), nickel (Ni), lead (Pb), manganese (Mn), and iron (Fe). The mineralisation of solid substrate was performed according to the standard ISO-1146: Soil quality—extraction of potentially toxic elements (PTEs) in aqua regia. To determine the content of most common cations and anions not bound interchangeably with the soil sorption complex, aqueous soil solutions with water-to-soil ratios of 5:1 were prepared, as described by Richards (1954). The suspensions were then filtered, and the filtrate was used to measure: content of Ca^2+^, Na^+^ and K^+^ with a flame photometer, and content of Mg^2+^ with an atomic absorption spectrophotometer. The resulting content of Na^+^ ions was used to calculate the sodium adsorption ratio (SAR), describing relative activity of sodium ions in exchange reactions according to the following formula:

$${\mathrm{SAR}} = \frac{{Na^ + }}{{\sqrt {\frac{{Ca^ + \,+ \,Mg^{2 + }}}{2}} }}$$

Soil productivity index (SPI) was calculated as proposed by Hu et al. (1992). The method was developed to assess soil quality, mainly in areas degraded by hard coal mining. It is based on an equation determining the relationship between the productivity index and the physical, chemical, and hydrological properties of soil. It takes into account the following soil properties: soil texture (granulometric composition), bulk density (g·cm^−3^), compactness (MPa), filtration coefficient (cm·h^−1^), easily available water content (%), infiltration (cm·h^−1^), porosity (%), soil pH, organic matter content (%), electrical conductivity (mS·cm^1^), root zone depth (cm), aggregate stability (%), sodium content, and stone share (%). All these parameters were used to calculate the sufficiency functions. SPI was calculated based on the following equation (Horn, 1971; Neill 1979; Hu et al. 1992):

$${\mathrm{SPI}} = \mathop {\sum}\limits_{i = 1}^n {\left( {w_i \times r_i} \right)}$$

where: SPI—soil productivity index, n—number of soil parameter, w_i_—weight coefficient for individual soil parameters, when:

$$\mathop {\sum}\limits_{i = 1}^n {W_i = 1}$$

r_i_—sufficiency coefficient for i-th soil parameter for a homogeneous soil profile.

Input data for the calculations were the determined properties of the material and assumed sufficiency functions ri, according to Hu et al. (1992). The receive input parameters grouped under the soil conditions aim at disentangling some mathematical aspects related to SPI for meaningful analysis. A further important aspect prior to proceeding with fuzzy set was selection of weight to make the number’s effect on the computation reflect its importance. As immediately appears, such coefficient (only the latter restricted number of factors) basically play the role in calculation by taking the lower reference category between elements of the dataset and values in the interval [0,1]. We applied 14 soil substrate parameters in the calculation of the SPI. Weight coefficients (wi) for almost all analysed properties were assumed to be 0.07, because of sum the weighting coefficients according to the equation should be 1. An exception was specific electrical conductivity, the values of which were higher than the limits for irrigated soils (Boroń et al. 2016). A weight coefficient of 0.09 was assumed for this parameter. As it follows from considerations concerning fuzzy data analysis, intermediate values cannot be equally important. Fundamental statistical requirement has been partly directly captured by a self-assessed variables considered sufficient for best representing the data. The use of homogeneous materials (such as sewage sludge mixtures) for reclamation purposes would not require separate expert opinions for various degraded soil.

### Fuzzy Set Theory and Statistical Analysis

An approach using fuzzy logic to determine the degree of degradation of the ideal solution was applied. The calculated sufficiency function was used in accordance with the rules of fuzzy logic. Fuzzy logic and fuzzy set theory are useful tools to calculate the uncertainty and inaccuracy of input data and they are described by means of a membership function. The fuzzy classification was determined by means of functions with groups of fuzzy classes (S).

$$S1,S2,...,Sk\left( {1 \;<\; k \;<\; n} \right)$$

In a fuzzy classification, an object (dataset) belongs to different fuzzy classes with different degrees of membership.

In our study, we used a fuzzy method of linear ordering and building a ranking of importance for each layer divided into: 0–20 and 20–50 cm. The parameters for each fuzzy number were estimated based on the minimum, dominant, and maximum values obtained for the test sample. The following conditions were met (Wolski, 2008).

$$0 \le fsj\left( {Pi} \right) \le 1\left( {i = 1,...,n;\,j = 1,...,k} \right)$$

where: j—indicates the number of fuzzy classes, f_sj_ (_Pi_)—designates the degree of association of _Pi_ object to S_j_ class:

$$\mathop {\sum}\limits_{j = 1}^k {fsj\left( {Pi} \right) = \left( {i = 1,...,n} \right)}$$

Loading factors obtained from a correlation matrix were included in factor analysis. Physical, chemical and hydrological properties of the initial soil (considered when describing its degradation degree) were verified by means of Detrended correspondence analysis (DCA) using Canoco 4.51 software to select the most important parameters. A hypothetical model was created consisting of two layers with thicknesses of 0–20 and 20–50 cm. The experimental data were divided into layers to compare the importance of soil properties of the tested Carbocrash substrate. Values exceeding 0.7 were marked in bold. Finally, only the values with the highest loading factors are shown in the ordination chart and Table 2. Multivariate regression was used in the assessment of predictors, and a model was built with Gretl statistical package version 1.9.9, however, only for the most important parameters. The results were verified with Multivariate analysis of the variance (MANOVA) approach using PAST software version 3.0.

Objects were classified to fuzzy classes based on the evaluation of depth intervals. Moreover, the usefulness of SPI was related to soil properties closely associated with plant growth and yield.

## Results

### The Height of Trees and Shrubs Measurement

In the study area, various species of trees and shrubs were evaluated. The dominant species among the trees was *Robinia pseudoacacia* (24) and *Betula verrucosa* (20), while among the shrubs it was *Elaeagnus angustifolia* (35) and *Tamarix parviflora* (17). The tallest of the *Robinia pseudoacacia* was 6.81 m, while *Betula verrucosa* reached a 6.42 m. The height of *Elaeagnus angustifolia* was estimated as 4.54 m, and *Tamarix parviflora* was 3.12 m. The tallest among *Hippophae rhamnoides* was 2.24 m. It should be noted that almost all of the invented trees and shrubs had good health. The DBH (1.3 m) measurements indicated that most of investigate plants were assigned a thickness class of 2–4 cm. Only trees and shrubs with a height of more than 150 cm were considered, therefore, *Pseudotsuga menziesi* and others were not included.

Plant species characterized by resistance to soil salinity were established on the plots, as evidenced by the intense dynamics of plant growth in subsequent vegetation seasons. The assessment of the woody and shrubs condition in the experimental plots showed relatively high growth. *Robinia pseudoacacia* and *Betula verrucosa* have revealed a fairly increase since the first plantings were made. Overall, it was found that the introduced revegetation method contributes to vigor plant health and appropriate growth parameters.

### Physical, Chemical, and Hydrological Properties of Soil

**Fig. 3:**
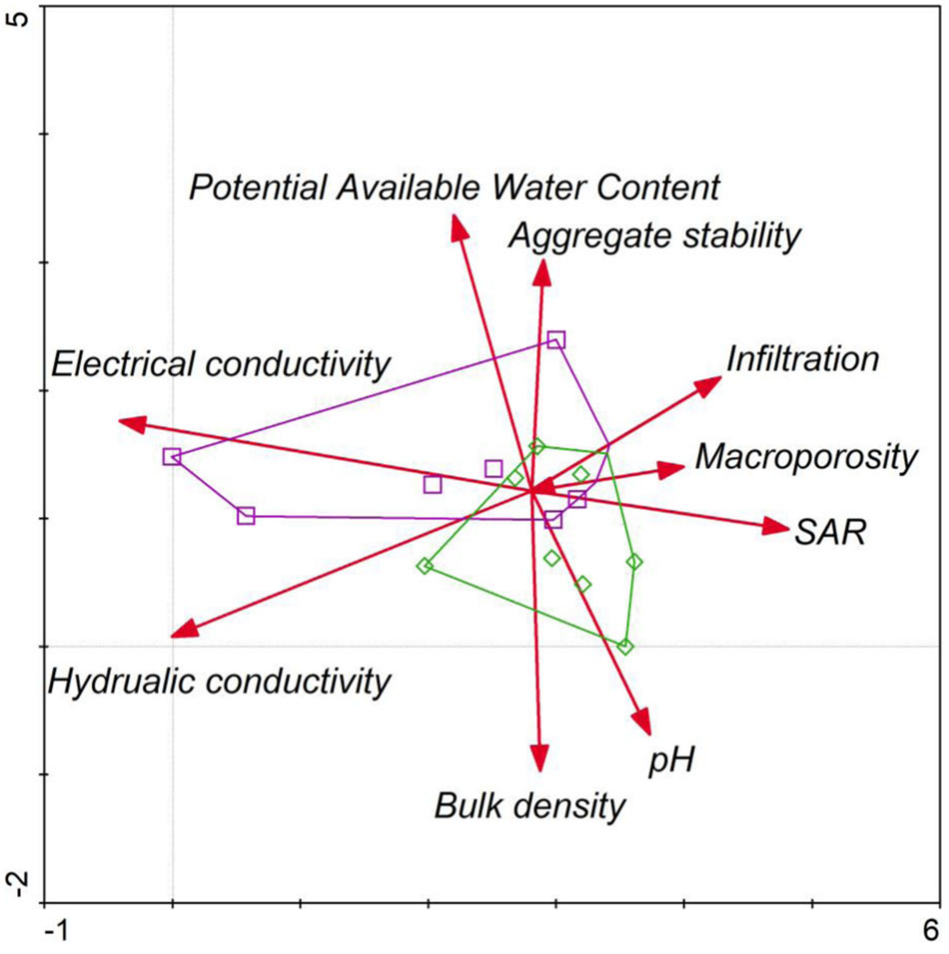
DCA plot. Open squares represent depth layer from 0–20 cm and diamonds indicate range between 20 and 50 cm depth. Eigenvectors designate factors affecting soil productivity by sewage sludge amendments. An analysis explained 68.2% of a total variance (42.1% for the first axis and 26.1% for the second one)

The basic physical and chemical parameters are presented in Table 1. A minimum thickness of reclamation cover is crucial for hydrological, drainage and maintenance purposes of the post-mining area. The results of the multivariate analysis (DCA) indicated that organic matter (*R*^2^ = 0.78) was the most important variable among the investigated parameters influencing SPI (Table 2). Infiltration negatively correlated with EC, while aggregate stability positively related with potential available water content, and there was a strong positive correlation between macroporosity and infiltration rate (Fig. 3). Factor loadings showed correlation affecting soil productivity by sewage sludge amendments (Table 3). The mean total organic carbon content for the Carbocrash substrate was 21.67%. The mean total nitrogen was 0.48%, and total phosphorus was 0.06%. Content of calcium was 0.44, and 0.43% for magnesium. In the case of sodium the mean content, with the values of 0.03%, and 0.71% for potassium were recorded. Volumetric water content at the point of field water capacity (pF = 2.5) was 0.39 cm^3^ cm^−3^ for the investigated Carbocrash substrate. Volumetric water content in the soil that was inhibiting plant growth (pF = 3.2) was 0.33 cm^3^ cm^−3^. At permanent wilting point (pF = 4.2) this value was ~0.27 cm^3^ cm^−3^. The supply of readily available water in the 50 cm layer was 2.53 cm, and total available water in this layer was 5.70 cm.

**Table 1:** Initial properties of the Carbocrash substrate

Variable	Unit	Mean ± SD
Bulk density	g cm^−3^	1.05 ± 0.29
Penetrometry	Mpa	0.69 ± 0.12
Infiltration rate	cm h^−1^	3.26 ± 0.98
Hydraulic conductivity (saturated)		1.51 ± 0.57
Electrical conductivity	dS m^−1^	1.51 ± 0.45
Sodicity	(SAR)	0.04 ± 0.01
pH	–	6.76 ± 1.52
Root media depth	cm	35.0 ± 5.78
Macroporosity	%	48.96 ± 4.69
Aggregate stability		38.5 ± 3.02
Stone content		20.0 ± 2.95
Organic matter		26.5 ± 4.01
Potential available water content		24.62 ± 2.76
Zn	mg kg^−1^	217.30 ± 32.23
Cu		42.09 ± 12.42
Ni		31.55 ± 1.53
Cd		0.62 ± 0.12
Pb		59.20 ± 2.23
Cr		42.15 ± 2.40
Mn		270.63 ± 29.24
Fe		874.28 ± 53.34

**Table 2:** Effects the most important Carbocrash substrate properties selected from DCA and predictors of multivariate regression model

Dependent variable	Regression summary		F1	F2	F3	F4
	*R*^2^	*p*	Potential available water content	Macroporosity	Infiltration rate	Aggregate stability
Organic matter	0.78	<0.01	0.17*	0.43**	0.40**	0.69
Root zone depth	0.75	<0.05	−0.22**	−0.63	0.78	0.57**
Bulk density	0.57	>0.05	0.84	0.76	−0.78	0.48
Depth (0–20 cm)	0.70	>0.05	0.60*	0.10	−0.54**	0.26
Depth (20–50 cm)	0.54	>0.05	−0.84	−0.15*	0.56	0.34

^*^P < 0.05; **P < 0.01

### Soil Productivity Index Based on Fuzzy Set theory

**Fig. 4:**
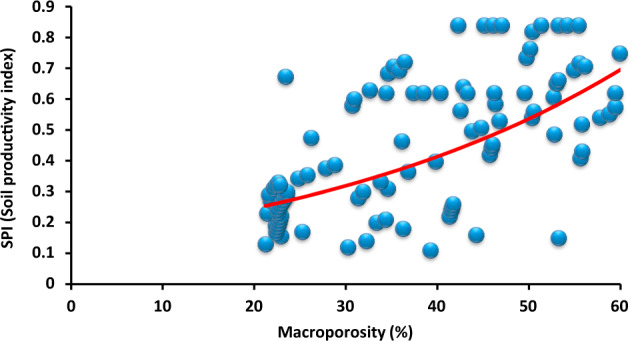
Relationship between soil productivity index and macroporosity

**Fig. 5:**
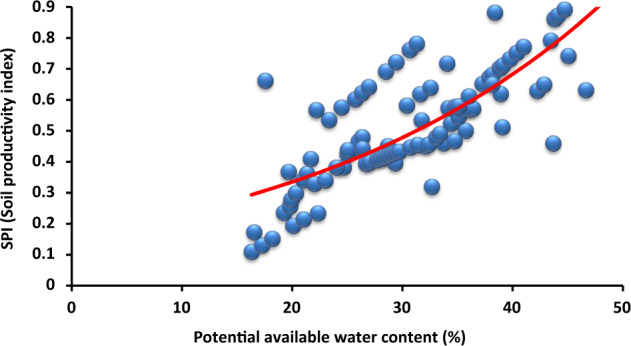
Relationship between soil productivity index and potential available water content

**Fig. 6:**
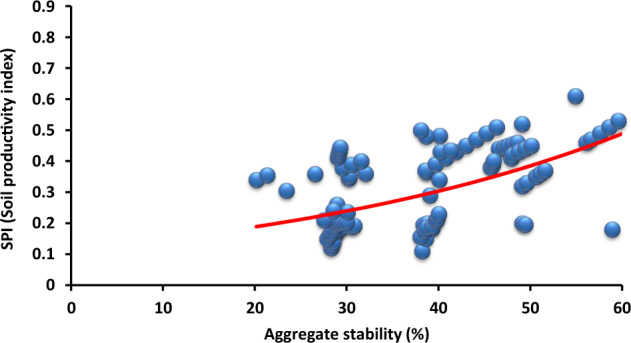
Relationship between soil productivity index and aggregate stability

The evaluation of Carbocrash substrate usefulness in the reclamation of post-industrial wasteland using fuzzy logic depends on the accuracy of selecting the form of membership function. The reclamation concept proposed in this paper was based on the function of depth intervals. The SPI determined for Carbocrash substrate was 0.81. Further, the properties of the reclaimed soil and waste used for its reclamation must complement each other, and create favourable conditions for plant growth and development. SPI based on the fuzzy set theory was very well suited for assessing degradation of the post-mining area only for macroporosity, potential available water content and aggregate stability (Figs. 4, 5, and 6).

## Discussion

### Physical and Chemical Properties of Carbocrash Substrate

Composition of the created initial soil was similar to that of sandy loam, and its hydraulic properties were suitable for plant growth. Further, organic matter content was particularly high (Table 1). Multivariate analysis demonstrated that generally available water, porosity, infiltration and aggregate stability were the most important among the investigated physical and chemical properties (Fig. 3). We recommend these parameters to be the first ones taken into account when assessing the degradation degree. Physical and chemical properties should be evaluated as the highest priority when introducing sewage sludge into the soil (Singh et al. 2011). The layers of 0–20 cm and 20–50 cm were important for multivariate regression, and differed significantly from the other layers. Infiltration is also essential. The F4 factor, in which infiltration was considered, had a well-adjusted regression model with the *R*^2^ coefficient equal 0.75 (Table 2). This demonstrated that water flow within the reclamation areas could affect other factors, and possibly limit them, particularly in deeper layers. Conversely, potential available water content was not dependent on any soil characteristic and varied weakly between 20 and 50 cm (Table 2).

In another study, the outcomes of a 10-year reclamation indicated that the average plant available water content in the first year after planting ranged between 2.3 and 5.9%. This was significantly higher than in the area of open pit coal mine reclaimed for a shorter time and in shallower layers (0–60 cm), where it ranged from 1.1 to 2.5% (Lei et al. 2016). The properties of the reclaimed soil and of the waste material used for this purpose may be assessed using the depth function, which evaluates active penetration of the root zone that provides soil protection. Our research implementation of this new remediation technology demonstrated that evaluation until 50 cm depth is necessary to plant development. Józefowska et al. (2017) claimed physical properties and biological conditions of reclaimed areas were appropriate at a depth of 0–20 cm. However, this conclusion might be misleading, as in degraded areas the physical, chemical, and hydraulic properties may vary at different depths. Our results indicated that after eight years of reclamation the 0–20 cm layer was different to deeper layer of 20–50 cm (Fig. 3). Moreover, in reclamation projects, the biological activity (Li et al. 2012) and fluctuations in physical and chemical properties should be conducive monitored over a long period of time (Juwarkar et al. 2010; Song et al. 2012; Helman et al. 2014; Huang et al. 2015).

### Challenges of Using Sewage Sludge in Biological Reclamation

Sewage sludge with amendments improving the chemical and physical properties, used for the reclamation of post-industrial wastelands, would contribute to the formation of better quality post-mining soils. Susceptibility of pollutants to degradation should be assessed in both physical and chemical terms prior to the implementation of bioremediation techniques (Kumpiene 2010). Other studies have provided further insights into the effects of intervention techniques. Fly ash and sewage sludge affects plants biomass, availability of nutrients, and soil properties (Tsadilas et al. 2014). Introduction of ashes into a sandy soil improves its structure and enhances its water capacity (Kumpiene et al. 2007). The mechanism of pollution retention by fly ash during soil reclamation involves mainly increasing soil pH, precipitation of pollutants and their sorption (Kumpiene 2010).

Data on electrical conductivity suggest that sewage sludge contains large amounts of dissolved salts. Further, the electrical conductivity of ash rock ranged from 1.12 to 1.28 mS cm^−1^ (Gilewska 2006). In our study, average electrical conductivity after 8 years of biological reclamation was 1.15 dS m^−1^. The most favourable changes in the investigated soil parameters were observed for high doses of sewage sludge, such as 40 and 60 t∙ha^−1^ (Antonkiewicz et al. 2016).

Our study showed that Carbocrash substrate could be useful in restoring the areas degraded by mining operations. This was indicated by the high content of organic matter and strong impact of potential available water content (Table 2). The use of biodegradable waste supplemented with Carbocrash substrate should be considered as improvements of the phytostabilisation process. An important international problem in degraded areas is exposure to heavy metals, and sewage sludge can have an impact in the accumulation of heavy metals in soil and plants (Kumar and Chopra 2014; Kumar et al. 2016). However, translocation of heavy metals from Carbocrash substrate to plants only presents a risk for Cd bioaccumulation (Halecki and Klatka 2017).

### Soil Productivity Index

Conventional methods of plant species selection prove to be insufficient (Ding et al. 2007; Zou et al. 2012). This is due to imprecise evaluation and not fully defined factors affecting the fertility of newly created initial soil. To overcome these obstacles, a multi-criterial decision method based on the fuzzy set theory was proposed. Average soil bulk density in our study was 1.05 mg m^−3^ (Table 1), and our analysis demonstrated its weak effects on hydraulic, physical and chemical properties (Fig. 3). However, results of the multivariate regression model (summarised in Table 2) verified that macroporosity tends to have a high positive correlation with bulk density. The criteria currently in use prevent reliable comparison of reclamation efficiency for different objects, or accurate assessment of applied reclamation methods (Halecki et al. 2016, Kim et al. 2018).

Our research indicated that the 8-year reclamation was the optimal way of managing mixtures containing Carbocrash substrate. Organic additives may enhance reclamation of contaminated soils, especially by improving physical properties around the plant root zone (Park et al. 2011). Further, the introduction of depth intervals for measuring individual parameters was important for the assessment of biological remediation. Hu et al. (1992) claimed the maximum value of the SPI to be 1.0. Moreover, an SPI ≤ 0.5 indicates strong degradation of soils, and an SPI > 0.8 is associated with low intensity of degradation processes. Our study demonstrated the most important physical properties of Carbocrash substrate in individual layers (Table 2). Soil productivity index has been associated with increased value of macroporosity (Fig. 4), potential available water content (Fig. 5), and aggregate stability (Fig. 6). Hence, we believe that accurate assessment of the materials used for reclamation of post-mining areas is the factor most profoundly affecting the effectiveness of projects aimed at improving productivity of post-industrial lands. The selection of proper soil properties, and the post-mining area, depends on numerous criteria and is a serious strategic challenge when deciding on a specific reclamation approach (Parraga-Aguado et al. 2014). Our study confirmed that not all of the factors contributing to soil properties need to be measured. Furthermore, root media depth was not a good predictor for the evaluation of a post-mine waste remediation procedure (Table 3).

**Table 3:** Factor loadings of Carbocrash substrate properties calculated by Detrended correspondence analysis

Variable	FC 1	FC 2	FC 3	FC 4
Bulk density	−0.22	−**0.73**	**0.78**	0.57
Penetrometery	0.60	0.10	0.54	0.26
Infiltration rate	0.57	−0.45	0.56	0.34
Electrical conductivity	**0.81**	−0.65	0.42	0.40
Hydraulic conductivity (saturated)	−0.52	−**0.86**	0.04	0.02
Sodicity	−0.56	0.69	0.39	0.18
pH	−0.46	0.27	0.17	0.40
Root zone depth	0.11	0.19	0.02	0.38
Macroporosity	0.46	0.54	0.15	0.39
Aggregate stability	0.63	0.58	0.22	0.07
Stone content	0.48	0.18	0.46	0.58
Organic matter	0.62	0.28	0.63	0.62
Potential available water content	**0.74**	−0.51	0.48	0.05

Indicators were ranked in ascending or descending order. The most important eigenvalues were bolded

FC means factor component

The suitability of Carbocrash, a substrate material used in this study, may be assessed based on depth function to determine the degree of root zone penetration. Water availability for plants depends on how deeply it is stored, its mobility, and the chemical composition of the soil. Elevated values of electrical conductivity recorded on experimental plots in the first years of the research period indicated high concentrations of soluble salts. This manifested due to the increasing osmotic pressure of the soil solution. It was concluded, therefore, that using Carbocrash substrate could result in limited water availability in the initial period, which highlighted the importance of irrigation. We assumed that current remediation strategies on rehabilitating areas must be widely discussed. Thus, appropriate studies aimed at partial, or complete management of the reclaimed areas are necessary.

## Conclusions

Eight years of reclamation with sewage sludge enriched with amendments was long enough to improve the physical properties of post-industrial soils. Uniform indicators would be a useful diagnostic tool in post-mining areas, especially in the context of monitoring changes in soil properties, development of habitats and potential productivity of plants. Soil substrate (“post-mining soil”) should be the basis for the diagnosis of habitats in reclaimed areas, and a desirable approach would be to identify indicators of soil evaluation in reclaimed areas. Reclamation of dumps does not fully account for specific conditions of these habitats, and does not always adjust the quality of applied methods to their specific requirements. Therefore, the evaluation of post-mining soils should account for increasing the significance of sewage sludge mixtures that improve the geomechanical properties of soil in a relatively short time. Further, the management of post-mining areas should focus primarily on biological reclamation with Carbocrash substrate, which is a highly effective substrate for post-mining soil. However, it is necessary to consider dynamic changes in the properties that are manifested during the reclamation procedure. The use of Carbocrash substrate improves the physical and chemical properties of initial soils in degraded areas. The productivity index was high in the areas reclaimed with Carbocrash substrate, which suggested a low level of soil degradation. The results of our study showed high usefulness of indicator methods based on properly constructed fuzzy sets and soil parameters. These would hugely simplify the reclamation technology, as they would require an assessment of soil productivity at the initial stage of reclamation for each post-mining area. Restoring functional quality and biological productivity might be faster with the use of Carbocrash substrate compared to mineral fertilisers. Environmental management systems should consider this biosolid in the integration of the reclamation process and improvement of hydraulic properties. Carbocrash substrate is especially recommended for the reclamation of soilless areas, coal-mining wasteland, landfills accepting metallurgical waste, and rock raw materials without humus layer. Introducing plant seedlings is a technical challenge of the reclamation process aimed at obtaining tree-covered land. This is why properly conducted remediation and revitalisation of post-mining areas should include profound changes in the assessment of plant production capacity within degraded areas. Further reclamation research should also take into account the phytoremediation ability of plants, which might accumulate contaminants from the post-mining areas. Studies and analyses investigating environmentally friendly methods of management of this innovative soil substrate will improve biological improvement of landfills, limit their negative impact, and reduce the amount of waste. This will facilitate proper shaping and protection of the natural environment.

## Data Availability

All data and materials as well as software application support our published claims and comply with field standards.
